# Ptch2 Deficiency Triggers Lipoma Formation and Adipogenic Transcriptome Reprogramming in Nile tilapia (*Oreochromis niloticus*)

**DOI:** 10.3390/ani16030405

**Published:** 2026-01-28

**Authors:** Changle Zhao, Xiang Liu, Xi Peng, Yongxun Chen, Shijian Peng, Lei Liu, Deshou Wang, Jing Wei

**Affiliations:** 1 Integrative Science Center of Germplasm Creation in Western China (CHONGQING) Science City, Key Laboratory of Freshwater Fish Reproduction and Development (Ministry of Education), Key Laboratory of Aquatic Science of Chongqing, Chongqing Technology Innovation Center of Breeding, School of Life Sciences, Southwest University, Chongqing 400715, China; changle0@email.swu.edu.cn (C.Z.); liuxiang1@email.swu.edu.cn (X.L.); zz1120243170001176@email.swu.edu.cn (Y.C.); fancyme@email.swu.edu.cn (S.P.); ll87v5@email.swu.edu.cn (L.L.); 2 Sichuan Industrial Institute of Antibiotics, School of Pharmacy, Chengdu University, Chengluo Avenue, Chengdu 610106, China; pengxi@cdu.edu.cn

**Keywords:** Patched2 (Ptch2), lipoma, Nile tilapia, transcriptome, lipid metabolism

## Abstract

The Hedgehog (Hh) signaling pathway is a key regulator of adipogenesis and lipid metabolism. However, the specific role of its receptor, Patched2 (Ptch2), in these processes remains unclear. Here, using a CRISPR/Cas9-mediated *ptch2* homozygous mutation model in Nile tilapia (*Oreochromis niloticus*), we found that Ptch2 deficiency induced visceral and perirenal lipomatosis characterized by small, multinucleated adipocytes. Comparative adipose transcriptomics revealed pronounced adipogenic reprogramming, with marked upregulation of genes governing de novo lipogenesis (e.g., *acaca*, *fasn*), fatty acid desaturation (e.g., *scd*, *fadsd6*), and triglyceride synthesis (e.g., *dgat2*, *lpl*). Biochemically, mutants exhibited elevated blood glucose and liver transaminases (alanine aminotransferase, aspartate aminotransferase) activity, and reduced alkaline phosphatase activity, indicating systemic metabolic dysregulation and hepatic stress. Our findings demonstrate that loss of Ptch2 triggers lipoma formation and adipogenic transcriptome reprogramming, highlighting its essential role in maintaining adipose tissue homeostasis.

## Simple Summary

This study explored the role of the gene *ptch2* in adipogenesis and lipid metabolism in Nile tilapia. Mutation of *ptch2* led to the formation of lipomas (benign fatty tumors) in the body cavity and around the kidneys. Analysis of fat tissue showed significant changes in gene expression, especially the upregulation of genes responsible for fat synthesis and storage. The mutant fish also exhibited higher blood glucose and signs of liver stress, indicating whole-body metabolic disturbances. These findings reveal that Ptch2 plays a key role in regulating normal fat tissue formation and lipid metabolism, providing a new model for studying related metabolic disorders.

## 1. Introduction

Adipose tissue is a dynamic endocrine organ primarily composed of adipocytes, serving as the central hub for systemic energy homeostasis through lipid storage and mobilization [1,2]. The differentiation and development of adipocytes, a process termed adipogenesis, are tightly regulated by complex transcriptional cascades [3,4]. Dysregulation of this process is implicated not only in prevalent metabolic diseases like obesity and type 2 diabetes but also in localized pathologies such as lipoma formation [5,6]. Lipoma is a benign mesenchymal tumor characterized by the uncontrolled growth of adipose tissue [7,8], and its pathogenesis is not yet clear.

Hedgehog (Hh) signaling pathway, a highly conserved pathway crucial for cell fate determination, tissue patterning, and stem cell regulation, is a potential regulator in adipose biology and tumorigenesis [9,10,11,12]. In canonical Hh signaling, the binding of ligands to the Patched (Ptch) receptor relieves its inhibition on Smoothened (Smo), ultimately activating Gli transcription factors [13,14]. In adipose biology, canonical Hh signaling acts as a potent inhibitor of white adipocyte differentiation [15]. In mice, the canonical Hh signaling, which is transduced via primary cilia, inhibits adipogenesis, whereas omega-3 fatty acids, which are sensed via the free fatty acid receptor 4 (FFAR4) in the cilium, promote adipogenesis [16,17]. In humans, Gli2-mediated Hh signaling is enriched in adipose progenitor cells and in dedifferentiated liposarcoma, where it promotes malignant progression by driving stromal formation and suppressing immune infiltration [18].

Patched2 (Ptch2) is a pivotal transmembrane receptor in the Hh pathway [13]. Ptch2 plays a crucial tumor-suppressive role in the development and progression of various cancers. In basal cell carcinoma, the absence of Ptch2 leads to abnormal activation of the Hh signaling pathway, which promotes tumor growth [19,20]. In breast cancer, truncating mutations in Ptch2 disrupt its transmembrane structure, activating the MAPK, PI3K/mTOR pathways and driving tumor cell proliferation [21,22]. These studies indicate that the normal function of Ptch2 is vital for inhibiting tumor development, and its mutations or deletions can activate oncogenic signaling pathways, thereby promoting tumorigenesis. However, the role of Ptch2 in lipomas remains elusive.

Nile tilapia (*Oreochromis niloticus*) is not only a valuable vertebrate model for genetic and physiological studies due to its well-annotated genome, suitability for CRISPR/Cas9-mediated genome editing [23,24], but also an important aquaculture species in which excessive visceral fat deposition commonly occurs, affecting growth efficiency and product quality [25]. This makes it a pertinent model for investigating the genetic regulation of adipogenesis and lipid metabolism. In this study, we systematically investigated the effects of Ptch2 deficiency on adipogenesis and lipid metabolism. Our results showed that Ptch2 deficiency leads to the formation of lipomas in Nile tilapia. Further histological and transcriptomic analyses demonstrated that Ptch2 acts as a key inhibitor of the adipogenesis program in vivo. These findings highlight a novel and critical role for Ptch2 in lipid metabolic homeostasis and tumor suppression.

## 2. Materials and Methods

### 2.1. Animals

Nile tilapias (*Oreochromis niloticus*) were reared in a recirculating freshwater system at 26 ± 0.5 °C under natural photoperiod. Water quality was monitored daily (pH: 7.2 ± 0.5; dissolved oxygen: 6.5–7.0 mg/L). Fish were fed three times daily with a commercial diet (Shengsuo, Yantai, China). All experimental fish were derived from crosses between XY males and XX females. Progeny were reared under the same conditions as parents. For phenotypic and molecular analyses, both male and female fish at 90 days after-hatching were used for all analyses. The selection of this time point is based on its correspondence to the post-juvenile growth phase in Nile tilapia [23]. All procedures complied with the Guide for Care and Use of Laboratory Animals and were approved by the Institutional Animal Care and Use Committee of Southwest University (NO. IACUC-20181015-12).

### 2.2. Cloning and Sequence Analysis

The putative coding sequence containing the complete open reading frame (ORF) of Nile tilapia *ptch2* was obtained through a search in GenBank (Gene ID: 100692939) and verified as follows. The sequence was amplified via RT-PCR using primers specific to *ptch2* (Table S1) under the following conditions: 95 °C for 3 min, 35 cycles of 95 °C for 30 s, 60 °C for 30 s, and 72 °C for 1 min, with a final extension at 72 °C for 10 min. The PCR product of the expected size was gel-purified, subcloned into the pMD19-T vector (Takara, Kusatsu, Japan), and confirmed by Sanger sequencing. Multiple amino acid sequence alignment and identity analysis were conducted using ClustalX 1.83 and GeneDoc 2.6 [26]. A neighbor-joining phylogenetic tree was constructed with MEGA 7.0 [27], and gene synteny analysis was performed using the Ensembl Genome Browser (http://www.ensembl.org). The domains of Nile tilapia Ptch2 were annotated based on the Patched receptor architecture in vertebrates [13], combined with validation through multiple sequence alignment with known orthologs.

### 2.3. Establishment of ptch2 Homozygous Mutant Lines by CRISPR/Cas9

The *ptch2*^−/−^ mutant line was established using the CRISPR/Cas9 system and hybridization, as described in our previous study [28]. The process encompassed target design, molecular cloning, microinjection, and genetic screening, as detailed below. First, the coding sequence of Nile tilapia *ptch2* (NCBI Gene ID: 100692939) was obtained from NCBI, and a 20-nt guide RNA (gRNA) target site within the first exon was selected to ensure disruption of the functional open reading frame. The target sequence (5′-GTCCCAGGGGCCGGCGTATT-3′) was identified using the online design tool ZiFiT (http://bio.tools/zifit, accessed on 25 January 2026). The primers listed in Table S1. The gRNA and Cas9 mRNA components were produced as previously described [24]. For microinjection, a mixture of gRNA (500 ng/μL) and Cas9 mRNA (1000 ng/μL) was co-injected into the freshly fertilized one-cell-stage embryos. Injected embryos were incubated in system water at 26 °C until hatching. The mutants were analyzed by polyacrylamide gelelectrophoresis (PAGE) and Sanger sequencing. The F0 chimeric adult XY males were crossed with wild-type (WT) XX females to obtain heterozygous F1 progeny. Then, at the same locus, the F1 siblings with 25 bp deletion in *ptch2* were intercrossed to obtain homozygous F2 mutants, respectively.

### 2.4. Hematoxylin and Eosin (H&E) Staining

Wild-type (WT) and *ptch2*^−/−^ fish at 90 dah were fixed in Bouin’s solution for 24 h at room temperature with agitation. The fixed samples were then processed as follows: serial dehydration in 60%, 70%, 80%, 90%, and 95% ethanol for 1 h each, followed by three 1 h immersions in 100% ethanol; sequential clearance in a xylene and ethanol mixture (1:1) for 30 min, followed by two 10 min immersions in xylene; and infiltration in paraffin for 2 h. The samples were sectioned at a thickness of 5 µm using a Leica Microsystems microtome (Wetzlar, Germany) and subsequently stained with H&E. Images were captured using an Olympus BX53 light microscope (Tokyo, Japan).

### 2.5. RNA-Seq

Adipose tissues were harvested separately from WT and *ptch2*^−/−^ fish at 90 dah, with three biological replicates performed for each group. Total RNA was extracted from each group using RNAiso Plus (Takara). RNA integrity was verified by agarose gel electrophoresis and quantified using a NanoDrop spectrophotometer (Thermo Fisher Scientific, Waltham, MA, USA). Libraries were constructed and sequenced on an Illumina NovaSeq 6000 platform (Illumina, San Diego, CA, USA) at GENEBOOK Biotechnology (Wuhan, China), generating 150 bp paired-end reads. Raw sequencing data were subjected to quality control using FastQC (v0.11.9) [29]. Clean reads from each library were aligned to the Nile tilapia reference genome (*Oreochromis niloticus* ASM185804v5) using HISAT2 (v2.2.1) [30]. The mapped reads for each sample were assembled, and transcript abundances were quantified using StringTie (v2.1.4) [31] in a reference-guided mode. Read counts mapped to each gene were calculated using featureCounts (v2.0.1) [32], and gene expression levels were normalized and reported as Fragments Per Kilobase of transcript per Million mapped reads (FPKM). Differentially expressed genes (DEGs) were analyzed with DESeq2 [33] and identified based on the following criteria: *p* value < 0.05 and |log2(fold change)| ≥ 1.5. Kyoto Encyclopedia of Genes and Genomes (KEGG) and Gene Ontology (GO) pathway enrichment analyses were conducted on the DEGs using an online analysis platform (https://www.genescloud.cn/login, accessed on 25 January 2026).

### 2.6. Quantitative Real-Time PCR (qRT-PCR)

Total RNA from the aforementioned samples was reverse-transcribed into complementary DNA (cDNA) using the PrimeScript II 1st Strand cDNA Synthesis Kit (Takara). Quantitative real-time PCR (qRT-PCR) was performed on an ABI-7500 system (Applied Biosystems, Waltham, MA, USA) following the SYBR^®^ Premix Ex TaqTM II (Takara Bio Inc., Kusatsu, Japan) protocol. Relative mRNA levels were normalized to *β-actin* and calculated using the formula R = 2^−ΔΔCt^ (primers listed in Table S1).

### 2.7. Serum Biochemistry

Blood was collected from the tail vein of WT and *ptch2*^−/−^ fish at 90 dah. Following collection, blood samples were allowed to clot at 4 °C for 2 h and then centrifuged at 3000× *g* for 15 min at 4 °C to obtain serum. Serum aliquots were stored at −80 °C until analysis. Levels of glucose, activities of alanine aminotransferase (ALT), aspartate aminotransferase (AST), alkaline phosphatase (ALP), total protein (TP), albumin (ALB), lipase, triglycerides (TG), total cholesterol (TC), high density lipoprotein cholesterol (HDL) and low-density lipoprotein cholesterol (LDL) were measured using the biochemical analyzer SMT-120VP (Seamaty, Chengdu, China).

### 2.8. Data Analysis

Statistical analyses were conducted using the GraphPad Prism 8 software package (GraphPad Software, La Jolla, CA, USA). Data are presented as mean ± SD derived from at least three independent experiments; each performed in triplicate. Statistical significance was assessed using a two-tailed Student’s *t*-test. The levels of statistical significance are indicated as follows: ** *p* < 0.01; * *p* < 0.05; NS, not significant.

## 3. Results

### 3.1. Sequence Analyses

**Figure 1:**
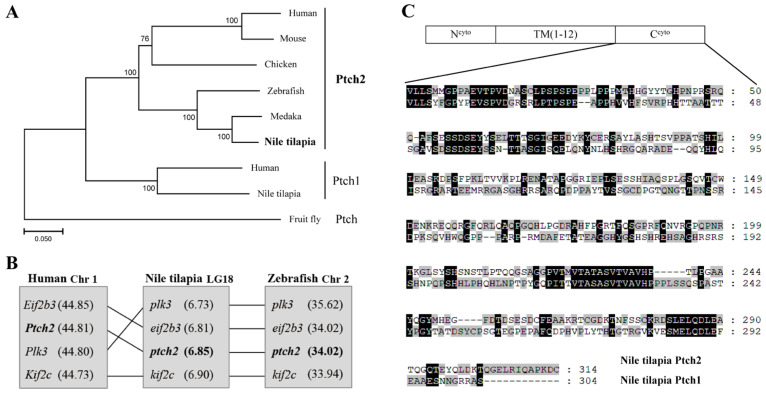
Sequence analysis of Nile tilapia Ptch2. (**A**) Phylogenetic analysis of Ptch proteins. The phylogenetic tree was constructed using the neighbor-joining method within the MEGA7.0 program. The length of the line is proportional to the evolutionary distance of the species to the branching point. Node values represent percent bootstrap confidence derived from 1000 replicates. Species-specific protein accession numbers are as follows: Human, *Homo sapiens*, PTCH2, NP 003729.3; Mouse, *Mus musculus*, Ptch2, NP 032984.1; Chicken, *Gallus gallus*, Ptch2, XP 025008840.1; Zebrafish, *Danio rerio*, Ptch2, NP 571063.2; Medaka, *Oryzias latipes*, Ptch2, XP_023820931.1; Nile tilapia, *Oreochromis niloticus*, Ptch2, XP 005476341.1; Human, PTCH1, NP 000255.2; Nile tilapia, Ptch1, XP 013127348.1; Fruit fly, *Drosophila melanogaster*, Ptch, NP 523661.2. (**B**) Chromosomal synteny analysis of the genomic region surrounding the ptch2 gene in Nile tilapia (LG18), zebrafish (Chr 2), and human (Chr 1). (**C**) Schematic representation of the domain structure of Nile tilapia Ptch1 and Ptch2, and sequence alignment of their C-terminal cytoplasmic domains (C^cyto^). N^cyto^: N-terminal cytoplasmic domain; TM: transmembrane domain; C^cyto^: C-terminal cytoplasmic domain. Amino acid numbers are indicated on the right. Dashes indicate deletions, and shaded areas indicate shared sequences.

The ORF of Nile tilapia *ptch2* was successfully cloned and validated. The resulting protein, composed of 1491 amino acids, exhibits a high sequence identity with vertebrate Ptch2 homologs, exceeding 65% (Table 1), and is phylogenetically clustered with these homologs, distinct from the Ptch1 clade and the *Drosophila* Ptch outgroup (Figure 1A). A comparative analysis of neighboring genes across species reveals that the chromosome 18, which contains *ptch2* in Nile tilapia, maintains good synteny with zebrafish chromosome 2 and human chromosome 1, both of which also harbor *Ptch2* (Figure 1B). Structural analysis indicates that Nile tilapia Ptch2 possesses characteristic domains, including a N-terminal cytoplasmic domain (N^cyto^) and 12 transmembrane domains, akin to its homolog Ptch1, although notable divergence is observed in the C-terminal cytoplasmic domains (C^cyto^) (Figure 1C). These findings suggest that Nile tilapia Ptch2 is orthologous to mammalian Ptch2, while exhibiting divergence from Ptch1.

### 3.2. ptch2 Deficiency Leads to Visceral and Perirenal Lipomatosis

**Figure 2:**
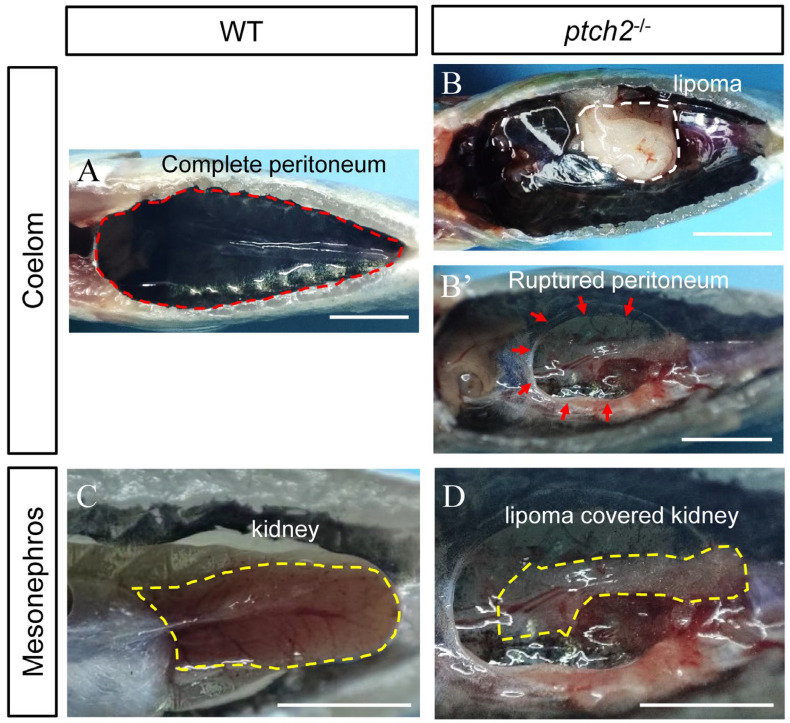
Phenotypes of WT and *ptch2*^−/−^ Nile tilapia at 90 dah. (**A**–**B’**) Coelom phenotypes of WT and *ptch2*^−/−^ fish after visceral removal at 90 dah. The red dashed line in (**A**) represents the intact peritoneum, the white dashed line in (**B**) represents the lipoma, and the red arrows in (**B’**) represents the site of the ruptured peritoneum. (**C**,**D**) Mesonephros phenotypes of WT and *ptch2*^−/−^ fish at 90 dah. The yellow dashed line in (**C**) represents a normal kidney, while the yellow dashed line in (**D**) represents a kidney wrapped with lipoma. Scale bars: 1 cm.

Homozygous *ptch2* mutants were viable at early stages but exhibited a striking internal phenotype by 90 dah. External morphology showed no significant difference from WT siblings. However, upon opening the body cavity, WT fish exhibited an intact peritoneum (Figure 2A), while *ptch2*^−/−^ mutants displayed prominent, encapsulated fatty masses (lipomas) attached to the inner body wall, with some causing visible rupture of the peritoneal lining (Figure 2B,B’). Furthermore, the kidneys of mutant fish were abnormally encapsulated by adipose tissue, unlike the clean surface of WT kidneys (Figure 2C,D). These results demonstrate that Ptch2 deficiency induces ectopic lipoma formation in the coelom and perirenal region.

**Figure 3:**
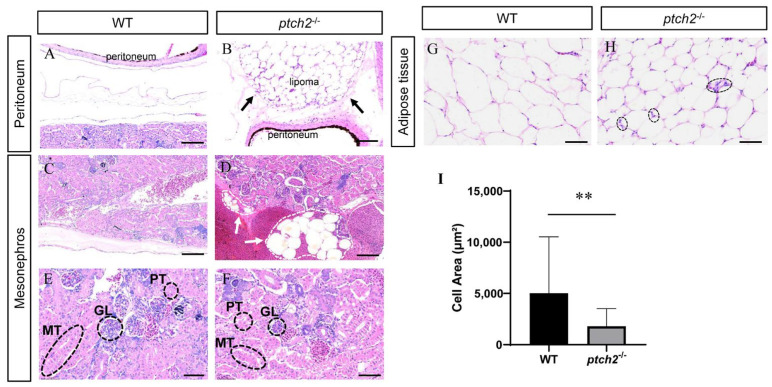
Histological analysis of coelomic tissues from WT and *ptch2*^−/−^ Nile tilapia at 90 dah. (**A**,**B**) H&E staining of peritoneal tissue. WT (**A**) shows normal peritoneal structure, whereas *ptch2*^−/−^ mutants (**B**) exhibit lipoma formation above the peritoneum (arrow). (**C**–**F**) H&E staining of Kidney tissue. WT (**C**,**E**) showing normal kidney structure. *ptch2*^−/−^ mutants showing normally in kidney tissue (**F**), but is covered with adipocyte clusters on the surface (arrows) (**D**). (**G**,**H**) H&E staining of adipose tissue. WT (**G**) showing typical adipose tissue morphology. *ptch2*^−/−^ mutants (**H**) showing smaller adipocytes and some cells with increased nuclearity (black dashed circles). (**I**) Statistical analysis of adipocyte area. Data are presented as mean ± SD. Significant differences between WT and *ptch2*^−/−^ groups were determined by Student’s *t*-test (** *p* < 0.01). GL, glomerulus; PT, anterior renal tubules; MT, mesonephric tubules. Scale bars, (**A**,**B**,**E**,**F**), 50 μm; (**C**,**D**,**G**,**H**), 100 μm.

Histological examination confirmed the macroscopic observations. H&E staining of peritoneal tissue from WT fish showed a normal, thin mesothelial layer (Figure 3A). In contrast, *ptch2*^−/−^ mutants exhibited a massive accumulation of adipocytes forming a discrete lipoma above the peritoneal layer (Figure 3B). The glomeruli, anterior renal tubules, and mesorenal tubules in the kidneys of WT fish developed normally (Figure 3C,E). Although kidney histology appeared normal in *ptch2*^−/−^ mutants (Figure 3F), the organ surface was covered by adipocyte clusters (Figure 3D).

Analysis of adipose tissue depots revealed a significant alteration in adipocyte morphology. Contrary to hypertrophy, adipocytes in *ptch2*^−^^/^^−^ fish were significantly smaller in area compared to those in WT (Figure 3G,H). Quantification confirmed a significant decrease in adipocyte area in mutants (Figure 3I). Notably, some mutant adipocytes contained increased nuclei within the lipid vacuole (Figure 3H), a feature occasionally associated with active adipogenesis or remodeling.

### 3.3. Transcriptomic Reprogramming in ptch2^−/−^ Adipose Tissue

**Figure 4:**
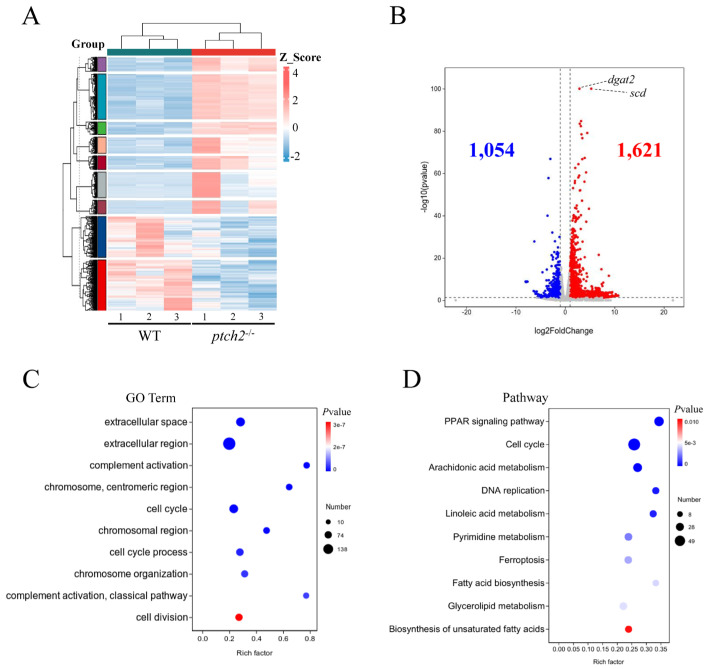
Transcriptome analysis of adipose tissue from WT and *ptch2*^−/−^ Nile tilapia at 90 dah. (**A**) Hierarchical clustering analysis of global gene expression patterns. Red indicates upregulated genes; blue indicates downregulated genes. (**B**) Volcano plot of differentially expressed genes (DEGs). A total of 1054 and 1621 genes were downregulated and upregulated, respectively, in the adipose tissue from *ptch2*^−/−^ mutants compared with the WT. (**C**) Scatter plot of the enriched GO terms for the DEGs. The sizes and colors of the dots represent the number of genes and the significance of the difference, respectively. (**D**) Scatter plot of the enriched KEGG pathways for the DEGs. The sizes and colors of the dots represent the number of genes and the significance of the difference, respectively.

To investigate the molecular basis of the phenotype, we performed RNA-seq on abdominal adipose tissue. Hierarchical clustering clearly separated WT and mutant samples (Figure 4A). We identified 2675 DEGs, with 1621 upregulated and 1054 downregulated in *ptch2*^−/−^ adipose tissue (Figure 4B). GO term enrichment analysis of DEGs revealed enrichment with biological processes such as “cell division” and “cell cycle” (Figure 4C). Consistently, KEGG pathway analysis showed significant enrichment for “PPAR signaling pathway”, “cell cycle”, “DNA replication”, “fatty acid biosynthesis”, “glycerolipid metabolism” and “biosynthesis of unsaturated fatty acids”. (Figure 4D). These data indicate a global transcriptional shift towards enhanced lipid synthesis and adipogenesis in the absence of *ptch2*.

**Figure 5:**
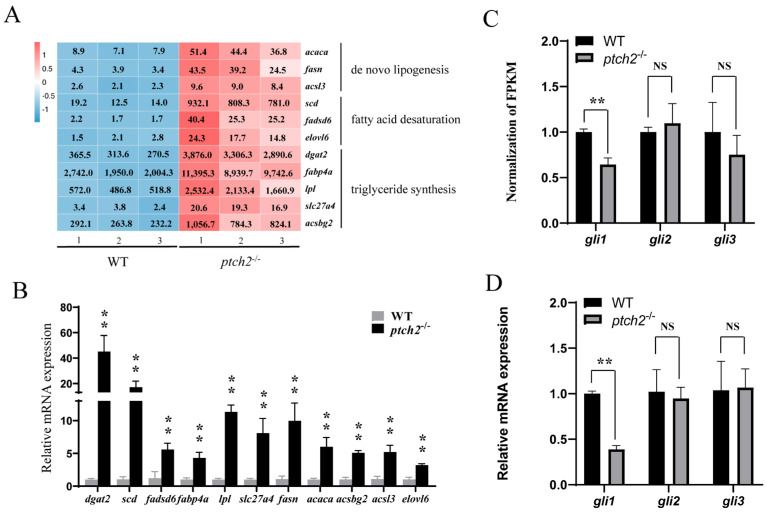
Expression of lipid synthesis-related genes and Hh signaling pathway genes in adipose tissue of WT and *ptch2*^−/−^ Nile tilapia at 90 dah. (**A**) Heatmap showing FPKM values of representative DEGs related to de novo lipogenesis (*acaca*, *fasn* and *acsl3*), fatty acid desaturation (*scd*, *fadsd6* and *elovl6*) and triglyceride synthesis (*dgat2*, *fabp4a*, *lpl*, *slc27a4* and *acsbg2*) from RNA-seq analysis. The number inside the square is the FPKM value of genes in each sample. (**B**) qRT-PCR validation of the lipogenic DEGs shown in (**A**). (**C**) FPKM values of Hh signaling pathway key genes *gli1*, *gli2 and gli3* from RNA-seq analysis. (**D**) qRT-PCR validation of the *gli1-3* expressions shown in (**C**). The *β-actin* gene was used as an internal control. Three biological replicates were analyzed, and significant differences were determined using Student’s *t*-test. The values are presented as the mean ± SD. **, *p* < 0.01. NS, not significant.

Focusing on the core lipogenic pathway, RNA-seq data showed a significant increase in the expression of key regulators and enzymes, including novo fatty acid synthesis (*acaca*, *fasn*, *acsl3*), fatty acid desaturation (*scd*, *fadsd6*, *elovl6*), and triglyceride synthesis (*dgat2*, *lpl*, *fabp4a*, *slc27a4*, *acsbg2*) (Figure 5A). This upregulation was independently validated by qRT-PCR, which confirmed a highly significant increase in the mRNA levels of these genes in *ptch2*^−^^/^^−^ adipose tissue compared to WT (Figure 5B). Meanwhile, we examined the expression levels of Hh signaling pathway key genes *gli1-3*. RNA-seq and qRT-PCR analysis showed that the expression of *gli1* was significantly downregulated in *ptch2*^−^^/^^−^ adipose tissue, while the expression levels of *gli2* and *gli3* remained unchanged (Figure 5C,D).

### 3.4. Systemic Metabolic Changes in ptch2 Mutants

**Figure 6:**
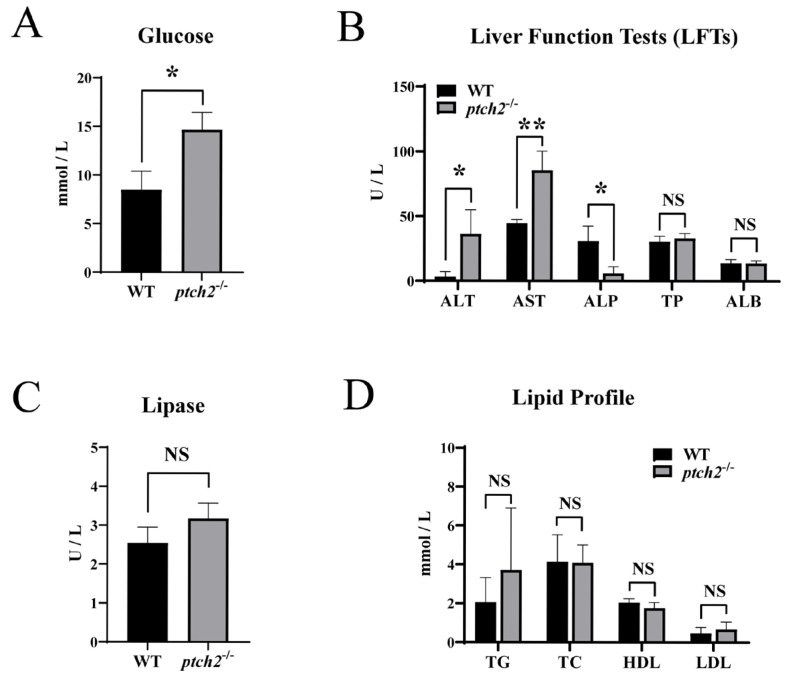
Serum biochemical parameters of WT and *ptch2*^−/−^ Nile tilapia at 90 dah. (**A**) Glucose levels. (**B**) Liver function tests: alanine aminotransferase (ALT), aspartate aminotransferase (AST), alkaline phosphatase (ALP), total protein (TP) and albumin (ALB). (**C**) Lipase levels. (**D**) Lipid profile: triglycerides (TG), total cholesterol (TC), high density lipoprotein cholesterol (HDL) and low density lipoprotein cholesterol (LDL). Data are presented as mean ± SD (*n* = 3). Significant differences between WT and *ptch2*^−/−^ groups were determined by Student’s *t*-test (* *p* < 0.05, ** *p* < 0.01). NS: not significant.

Serum biochemical analysis showed that *ptch2*^−/−^ mutants had elevated blood glucose levels (Figure 6A) and increased activities of alanine aminotransferase (ALT) and aspartate aminotransferase (AST), along with reduced alkaline phosphatase (ALP) activity (Figure 6B). No significant changes were observed in total protein, albumin, lipase, or lipid profiles (triglycerides, total cholesterol, HDL, LDL) (Figure 6B–D).

## 4. Discussion

This study reveals that Ptch2 deficiency in Nile tilapia results in a distinct pathological syndrome characterized by visceral lipomatosis and systemic metabolic dysregulation. The formation of coelomic and perirenal lipomas, underscores a critical role for Ptch2 in maintaining adipose tissue homeostasis.

Several lines of evidence establish the Hh signaling pathway as a potent inhibitor of adipocyte differentiation and terminal maturation. In vitro, activation of Hh signaling in human mesenchymal stem cells impairs adipocyte maturation, resulting in smaller cells with reduced lipid droplet size [34]. Similarly, Sonic Hh promotes osteoblastic commitment while suppressing adipocytic differentiation in pluripotent mesenchymal cells [35]. In vivo, supporting this inhibitory role, adult mice bearing a truncated Ptch1—which leads to constitutive Hh pathway activation—exhibit reduced white fat mass, smaller adipocytes, and downregulation of adipocyte marker genes (e.g., aP2, adipsin) [15]. Structurally, Ptch2 shares conserved core domains with Ptch1, including a N-terminal cytoplasmic domain and 12 transmembrane domains, yet they diverge notably in their C-terminal cytoplasmic tails. Our previous study suggests that ptch1 mutation in Nile tilapia causes dying during the 6–8 days post fertilization [36], precluding the study of its role in postnatal adipose biology. Our work establishes that Ptch2, in contrast, is essential for postnatal adipose homeostasis. *ptch2* mutant tilapias develop lipomas composed of densely packed, small, and multinucleated adipocytes (Figure 3G–I), demonstrating that Ptch2 acts as a non-redundant, indispensable suppressor of pathological adipo-genesis in vivo.

Transcriptomic profiling of adipose tissue from *ptch2* mutants revealed extensive transcriptional reprogramming, with over 2,600 DEGs. KEGG analysis indicated significant enrichment in pathways related to lipid metabolism, including “Fatty acid biosynthesis,” “Glycerolipid metabolism,” and “Biosynthesis of unsaturated fatty acids” (Figure 4D). Notably, the PPAR (peroxisome proliferator-activated receptor) signaling pathway—was among the most prominently upregulated. This pathway coordinates adipocyte differentiation, lipid synthesis, and metabolism through a network of downstream targets. Key PPAR responsive genes such as *fabp4a*, *scd*, *fadsd6*, *lpl*, and *slc27a4* were consistently up-regulated in the mutant lipomas (Figure 5).

The induction of *fabp4a* is particularly notable: its encoded protein, fatty acid binding protein 4, not only serves as a direct transcriptional target of PPARγ but also enhances PPARγ transactivation, establishing a potent feed forward loop that drives adipogenic commitment [37]. Similarly, SCD1 (stearoyl CoA desaturase 1) catalyzes monounsaturated fatty acid synthesis under PPAR regulation, and its loss has been shown to redirect lipid metabolism via PPARβ dependent rewiring [38]. SLC27A4 (fatty acid transport protein 4), which mediates cellular fatty acid uptake, is transcriptionally activated by PPARα, highlighting the pathway’s role in governing lipid import [39]. Together, this coordinated transcriptional activation provides a molecular basis for the neoplastic phenotype observed—characterized by proliferating small adipocytes—rather than simple adipocyte hypertrophy, and directly links Ptch2 deficiency to a pro adipogenic PPAR driven program.

The significantly reduced survival of *ptch2*^−/−^ mutants (Figure S1) likely stems from a combination of anatomical and systemic pathological insults. The massive coelomic lipomas physically displace and compress vital organs, most notably the kidneys (Figure 2B,B’,D), potentially compromising their architecture and function. Concurrently, the local disruption of adipose homeostasis triggers severe systemic metabolic dysregulation. Marked hyperglycemia (Figure 6A) suggests the systemic glucose intolerance, a common sequela of dysfunctional adipose tissue [40]. Elevated levels of hepatic transaminases (AST, ALT; Figure 6B) indicate secondary hepatic stress [41], likely resulting from altered systemic nutrient flux or inflammatory signals originating from the pathological fat depots. Thus, we propose that the observed lethality of *ptch2*^−/−^ mutants may be attributed to the compounded effects of mechanical organ compromise and pervasive metabolic dysfunction. Future studies are warranted to delineate the precise contribution of each factor.

Surprisingly, analysis of key Gli transcription factors in mutant adipose tissue revealed a significant downregulation of *gli1*, with no significant change in *gli2* or *gli3* expression (Figure 5C,D). This pattern is paradoxical within the canonical Hh signaling framework, where loss of the inhibitory receptor Ptch2 would be predicted to derepress the pathway and increase Gli activity [9]. This paradoxical observation aligns with emerging evidence for context-dependent complexity in Hh pathway regulation. For instance, the core cytoplasmic suppressor Sufu is a well-established negative regulator; however, its genetic ablation can produce opposing effects in a tissue-specific manner. In neural stem cells of the dentate gyrus, loss of Sufu reduces downstream Gli1 target expression, contrasting with its effect in the neocortex where Sufu deletion increases signaling, highlighting its context-dependent role [42,43]. This provides a conceptual parallel, suggesting that Ptch2 deficiency may not result in a straightforward hyperactivation of canonical signaling but is more likely to trigger a tissue-specific rewiring of the pathway. In this specific context, Ptch2 may play a non-canonical role in stabilizing transcriptional complexes or balancing the activities of different Gli factors, with its loss ultimately leading to the dysregulation of adipogenic transcriptional programs. Future studies are needed to elucidate the precise molecular mechanism by which Ptch2 loss leads to Gli1 downregulation in adipose tissue and to define how this rewired signaling state mechanistically drives lipoma formation.

## 5. Conclusions

In summary, our results delineate a novel pathway in which Ptch2 acts as a guardian of adipose tissue integrity. Its deficiency initiates a transcriptional cascade through the activation of the PPAR pathway, resulting in lipomatous transformation and subsequent metabolic dysfunction. This study not only identifies Ptch2 as a critical regulator of fat biology but also establishes a robust aquatic model for dissecting the mechanisms of adipogenesis and systemic fat metabolic comorbidities.

## Figures and Tables

**Table 1:** Amino acid sequence identity of Ptch among different species (%).

Species	Full Length	Domain		
		N^cyto^	TM (1–12)	C^cyto^
Ptch2 (Nile tilapia)	100	100	100	100
Ptch2 (Zebrafish)	80	90	88	51
PTCH2 (Human)	65	56	67	44
Ptch1 (Nile tilapia)	62	67	61	29

N^cyto^: N-terminal cytoplasmic domain; TM: transmembrane domain; C^cyto^: C-terminal cytoplasmic domain.

## Data Availability

The data that support the findings of this study are available from the corresponding author upon reasonable request.

## Supplementary Materials

The following supporting information can be downloaded at: https://www.mdpi.com/article/10.3390/ani16030405/s1, Figure S1. Genotype distribution in surviving offspring from *ptch2*^+^^/−^ intercrosses at different developmental stages. Table S1. Sequences of the primers used in this study.
