# Supplementary material for: Ptch2 Deficiency Triggers Lipoma Formation and Adipogenic Transcriptome Reprogramming in Nile tilapia (Oreochromis niloticus)
Source: Animals (Basel). 2026 Jan 28;16(3):405. doi: 10.3390/ani16030405 (PMC12896722; doi:10.3390/ani16030405)
Supplement: Supplementary file 1 [file animals-16-00405-s001.zip › Figure S1.pdf]

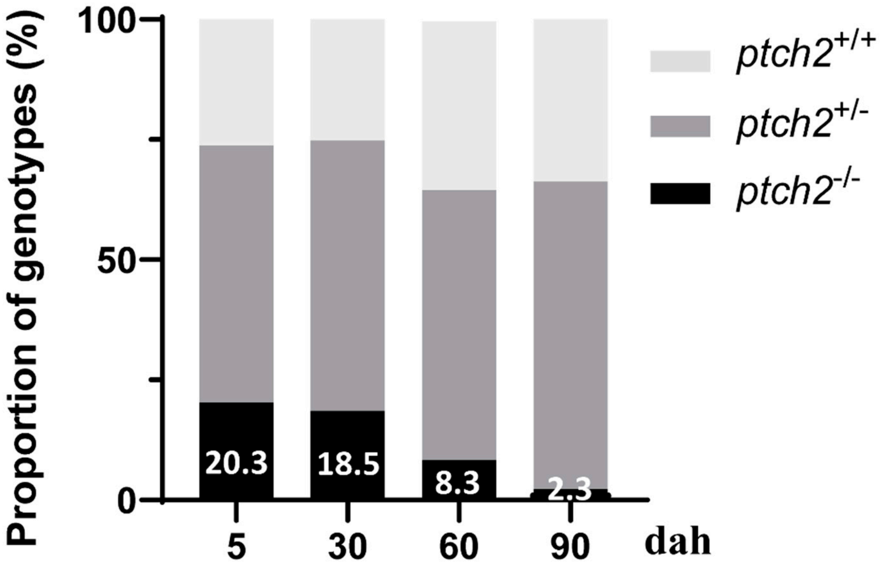

Figure S1. Genotype distribution in surviving offspring from *ptch2*$^{+/-}$ intercrosses at different developmental stages. Proportion of *ptch2*$^{+/+}$, *ptch2*$^{+/-}$ and *ptch2*$^{-/-}$ fish at 5, 30, 60 and 90 dah. The white numbers in the figure represent the survival rate of *ptch2*$^{-/-}$ fish at each time point. Genotype distribution for each genotype: 5 dah: *ptch2*$^{+/+}$ (53/202), *ptch2*$^{+/-}$ (108/202), *ptch2*$^{-/-}$ (41/202); 30 dah: *ptch2*$^{+/+}$ (56/222), *ptch2*$^{+/-}$ (125/222), *ptch2*$^{-/-}$ (41/222); 60 dah: *ptch2*$^{+/+}$ (106/302), *ptch2*$^{+/-}$ (171/302), *ptch2*$^{-/-}$ (25/302); 90 dah: *ptch2*$^{+/+}$ (60/178), *ptch2*$^{+/-}$ (114/178), *ptch2*$^{-/-}$ (4/178).
